# EMG Characterization and Processing in Production Engineering

**DOI:** 10.3390/ma13245815

**Published:** 2020-12-20

**Authors:** Manuel del Olmo, Rosario Domingo

**Affiliations:** Department of Construction and Manufacturing Engineering, Universidad Nacional de Educación a Distancia (UNED), C/Juan del Rosal 12, 28040 Madrid, Spain; mdelolmo36@alumno.uned.es

**Keywords:** EMG, production engineering, human-machine interaction, monitoring, ergonomics

## Abstract

Electromyography (EMG) signals are biomedical signals that measure electrical currents generated during muscle contraction. These signals are strongly influenced by physiological and anatomical characteristics of the muscles and represent the neuromuscular activities of the human body. The evolution of EMG analysis and acquisition techniques makes this technology more reliable for production engineering applications, overcoming some of its inherent issues. Taking as an example, the fatigue monitoring of workers as well as enriched human–machine interaction (HMI) systems used in collaborative tasks are now possible with this technology. The main objective of this research is to evaluate the current implementation of EMG technology within production engineering, its weaknesses, opportunities, and synergies with other technologies, with the aim of developing more natural and efficient HMI systems that could improve the safety and productivity within production environments.

## 1. Introduction

Electromyographic (EMG) signals are biomedical signals that measure the electrical current generated during muscle contractions. These signals represent neuromuscular activity and are dependent on the anatomical and physiological characteristics of the muscle. Nowadays, EMG has many applications, including the analysis of neuromusculoskeletal diseases, orthosis control, or ergonomic assessment. However, EMG technologies present some limitations that prevent their use beyond research and clinical environments.

The evolution of EMG signal analysis techniques combined with the development of new instrumentation has great potential in production engineering: fatigue monitoring of workers—based on live musculoskeletal risk assessment associated with an activity—or the development of new human–machine interaction systems, including remote control of robotic arms and other industrial equipment or the prediction of movements of workers, are now feasible technologies. There are still many challenges to overcome for the effective use of this technology to offer new possibilities in the future. The digital development of the industry, which is now capable of generating and processing an enormous amount of data, will undoubtedly be benefited from techniques such as massive data analysis and artificial intelligence, which when applied to the analysis of biomedical signals will transform the human being into an active element of the production processes in an evolved and completely different way from what is known.

In this paper, a state-of-the-art review of current applications of EMG techniques applied to production engineering is presented. After analyzing EMG signal acquisition and processing techniques, successful production engineering EMG cases of use are reviewed. Trends, synergies with other technologies, opportunities, and limitations are identified, establishing a compendium of knowledge to allow the improvement of safety and productivity within production environments.

## 2. Electromyography

The first reference of muscle generated electricity dates from 1666; Francesco Redi suspected that the discharge produced by an electric fish was of muscular origin. However, it was not until 1791 that Luiggi Galvani (1737–1798), professor of anatomy at the University of Bologna, recognized the relationship between the electrical stimulation of a nerve and the contraction that is generated in a muscle [1]. He developed the concept of “*animal electricity*”, which refers to the generation of electricity within the body that is subsequently channeled through the nervous tissue [2] and a concept that is reflected in his book *De Viribus Electricitatis* [3]. After his discoveries, a strong scientific controversy followed in Italy between Galvani and Alessandro Volta (1755–1832), who questioned the results obtained by Galvani [4]. In 1838, Carlo Matteucci (1811–1868) succeeded in proving that the electric current described by Galvani is truly generated by the muscles. However, it was not until 1849 that the first electrical signal generated by a muscle due to a voluntary contraction was detected by Emil Du Bois Reymond (1818–1896) [3].

The evolution of electromyography has been possible due to the evolution of acquisition equipment and processing techniques. Ferdinand Braun developed in 1897 cathode-ray tubes, which were used with a string galvanometer by Forbes and Thatcher in 1920 [5] to measure EMG signals. Gasser and Erlanger [6] used an oscilloscopic instead of a galvanometer two years later, showing for the first time EMG signals using one single piece of equipment. Piper used a metal surface electrode for the first time in 1907, simplifying the process of measuring the human muscle contractions. He presented his experiments and results in the first book that addresses the subject of Electromyography in 1912, “*Elektrophysiologie menschlicher Muskeln*”.

Edgar Douglas Adrian (1889–1977), the winner of the Nobel Prize in Medicine in 1932 for his discoveries related to the functioning of neurons, collaborated with the American physiologist Detlef Bronk in an experiment to quantify the action generated by a single nerve fiber. They used a needle electrode and a speaker to record the electrical activity of muscle fibers that are generated through a single nerve fiber, which would later be called "Motor Potential Unit". The electromyogram obtained from a human tricep is composed of “rhythmic discharges of different groups of muscle fibers” [7] that vary in frequency with the force of the contraction and responding different groups of fibers in different phases, which causes high-frequency oscillations.

Fritz Buchthal (1907–2003) developed a microelectrode in 1934 to record the potential of isolated muscle fibers. One of his greatest achievements was to develop the normative data used today regarding the amplitude and duration of motor potential units in different muscles and ages between the years 1954 and 1955. Edward H. Lambert (1915–2003) established the first clinical electromyography laboratory in the United States in 1943 upon joining the Mayo Clinic after earning his PhD from the University of Illinois. Years later and after numerous studies, James A. Fizzel, Herbert Jasper, and James Golshet designed and developed the first commercially viable electromyography equipment for medical use in 1948.

The electromyography is rapidly adapted to the clinical field, so standardization and improved communication between researchers and professionals in the sector is necessary. In August 1953, the American Association of Electromyography and Electrodiagnosis was created in Chicago, developing the first scientific seminar in September 1954 [1].

Erik Stålberg, the author of the book Single Fiber Electromyography [8] in 1979, began to study the speed of propagation of electrical impulses towards the muscle fibers. Based on Buchthal’s studies, he manages to develop a smaller electrode using suitable amplifiers and filters. This new development allows the users to record a single muscle fiber.

The development of electromyography has been possible thanks to the contribution of many scientists and professionals from different fields, both clinical and engineering. Although its foundations have been known for many years, the evolution it has had in the last century has been possible thanks to the technological advancement of instrumentation, which still has several drawbacks that make it difficult to use. Sensors, data processing, and other features will need to continue evolving so that in the future, we can see this technology fully integrated into society and industry.

### 2.1. Muscle Contractions

Muscle contraction is the physiological process by which a muscle shortens or relaxes due to the sliding of the internal structures that compose it. An action potential is a wave of an electrical discharge that passes through a cell membrane and modifies its polarity by altering the concentration of sodium, potassium, and calcium ions inside and outside the cells. In general, the muscle contraction process begins with the action potential initiated at the neuromuscular junction, which spreads to the muscle fibers and T-tubules.

During an action potential, the inflow of sodium causes the depolarizing phase of an action potential, whilst an outflow of potassium ions causes its repolarizing. The inflow of sodium within a cell changes the membrane potential during an action potential from −55 to +30 mV. Calcium required to activate contractile proteins is stored in bag-shaped structures called sarcoplasmic reticules, whose function is to measure the concentration of calcium ions in the sarcolemma through its release. When crossing an action potential, the T-tubules produce an interaction with the sarcoplasmic reticulum, which produces a release of calcium ions and initiates the interaction between myosin and actin, finally producing muscle contraction [9,10].

Muscle contractions include four different phases: ATP hydrolysis that reorients and energizes the myosin head, the attachment of myosin to actin to form cross-bridges, the power stroke produced as the cross-bridges rotates toward the center of the sarcomere and the detachment of myosin from actin after the power stroke [10].

### 2.2. EMG Signal Structure

The action potential of a muscle fiber (MFAP) is the fundamental component of an electromyographic signal. It corresponds to the potential action that crosses a muscle fiber that belongs to a motor unit (MU). The characteristics of the MFAP will depend on the diameter of the fiber, the conduction speed, and its relative position with respect to the electrode that acquires the signal [11].

A group of muscle fibers innervated by a single motor neuron is called a motor unit, being the minimum functional unit that can be controlled by neuronal action. Muscle force is produced by activating the motor neuron, which causes tension in the associated fibers and produces a muscle contraction. When the activity of the motor neuron ceases, muscle relaxation occurs.

The motor unit action potential (MUAP) is the sum of the action potential of muscle fiber contractions in a motor unit, which can be measured by surface electrodes or sensors. The voltage detected by the sensors represents the summation of the activity of all active motor units. Those MU with a closer proximity to the electrode will be detected easily, causing attenuation and greater noise in more distant MU. The aggrupation of MUAP*s* generated from a single MU is noted as motor unit action potential train, MUAPT. The EMG signal obtained from a single electrode in a single contraction could be represented as follows [12]:

$${EMG}_{t}\;=\;\sum_{m\;=\;1}^{N}MUAPT_{m}\left(t\right)+n\left(t\right)$$

In this expression, $MUAPT_{m}\left(t\right)$ represents the temporal function of the voltage contribution of a MUAPT *m* of *N* motor units that contributes to the signal. The function *n*(*t*) represents all the components of the signal not associated with the motor unit potential, being environmental and biological noises. The characteristics of this signal are highly dependent on the level and duration of contraction, dynamic, and static state of the muscle, fatigue, and skin sweat. Using needle electrodes that can be placed close to a single muscle fiber, the electrical activity of that fiber can be detected, identifying the activity of a single motor unit. On the contrary, using surface electrodes, the detected signal will be composed of MUAPTs of various motor units, not being able to discriminate the origin of each signal component.

### 2.3. Acquisition

The acquisition of EMG signals is carried out by electrodes that acquire the electrical potential of muscles during contraction and are positioned in contact with muscle fibers or the skin. Two general types of electrodes could be identified: surface and needle electrodes.

Needle electrodes could acquire EMG signals from a single muscle fiber with high precision, but their invasive nature is a major drawback, making them unviable for any commercial application. Surface electrodes acquire EMG signals from several motor units, obtaining noisy signals. Two types of surface electrodes could be identified: dry and wet electrodes. Wet electrodes require a layer of conductive gel between the skin and the metal part, while surface electrodes will be in direct contact with the skin. Both types of electrodes can achieve equivalent signal quality [13], which makes dry electrodes more suitable for industrial applications. Novel types of dry electrodes based on architecture titanium thin films [14] or flexible hydrographic printed dry electrodes [15], that may be attached to the body similar to a tattoo, offer an alternative to conventional wet Ag/AgCl electrodes with improved biocompatibility, usability, and long-term wearability. Recently, conductive hydrogels electrodes have been developed to capture biosignals, such as EMG or ECG (ElectroCardoGram), accurately [16].

Different electrode configurations are considered for the acquisition of EMG signals: monopolar, bipolar, and Laplacian [17]. The Laplacian configuration is the configuration less susceptible to noise generated by nearby muscle activity [18]. High-density surface electrodes, also known as HD-sEMG electrodes, form a grid of electrodes that could acquire the EMG signals originated in the entire area of interaction of the muscle with the skin [19], easing the spatial analysis of muscle behavior. The use of HD-sEMG sensors allows the development of new analysis models to estimate non-superficial muscle activation. Piovesan et al. [20] evaluate the use of graph theory and electrical networks to recognize forearm muscle patterns corresponding to a single section of the forearm, obtaining promising results. TeleMyo™ Clinical DTS (Noraxon, Scottsdale, AZ, USA) [21], FreeeEMG (BTS Bioingenieering, Quincy, MA, USA) [22], and Myo (Myontec, Kuopio, Finland) [23] are commercial devices used to acquire surface electromyography (sEMG) signals. In addition, open-source hardware such as Arduino (Arduino, Ivrea, Italy) [24] and Raspberry (Raspberry Pi, Cambridge, UK) [25] could also be used to acquire sEMG signals [26].

Electromyography technology has some limitations that prevent its full implementation in applications beyond the clinical field, such as industry. The equipment used for the acquisition of EMG signals, especially in real-time commercial applications, needs to fulfill some important characteristics: to be simple, intuitive, portable, cheap, reliable, and robust [27].

One of the most recurrent problems is the displacement or loss of the electrode from the analyzed muscle, which causes noise or the total loss of the signal. In this regard, needle sensors are not appropriate for commercial applications, as they cause discomfort to the user despite avoiding the displacement of the sensors. New high-density surface electromyography sensors, HD-sEMG, have been developed for signal acquisition. The use of these sensors allows the processing of the entire surface of the interaction between muscle and skin, so the noise caused by its displacement during use can be solved by reconstructing the interaction map using algorithms.

A displacement in the position of the electrodes causes a considerable reduction in the performance of the identification system, as the new signals obtained from the sensors are different from those used during the learning process. To solve this problem, different alternatives have been evaluated from the point of view of data processing such as using data from displaced sites in the learning process [28] or evaluating the use of parameters invariant to sensor displacement [29]. Recently, He et al. [30] propose a user-oriented evaluation framework to determine if the sensors are in a position within the allowed tolerance, enabling a prior adjustment to the recognition process.

In addition to a possible displacement of the sensor during use, one of the existing limitations is the required contact of the electrode with the skin. The development of integrated sensors in textile products for the acquisition of biomedical signals allows the acquisition of data in a more realistic, casual, and outside laboratory conditions [31]. As an example, the use of compression clothing with integrated EMG sensors for athlete training and ergonomic evaluation has recently been commercialized by several companies, such as Mad Apparel Inc. (San Jose, CA, USA) [32] and Myontec Ltd. (Kuopio, Finland) [33].

## 3. Signal Analysis

### 3.1. Sampling, Filtering, and Segmentation

The objective of processing and segmenting the EMG signal is to adapt it for later analysis, eliminating the noise that might lead to wrong interpretations.

Ratio and resolution should be considered in EMG signal sampling [34]. Most of the power of an EMG signal is below 400–500 Hz, so according to Nyquist–Shannon sampling theorem [35], sampling rates above 1 KHz should not be considered. Some authors have evaluated lower sampling rates, obtaining a significant reduction of resources required during signal processing with a slight reliability reduction on hand movements classification [36].

The use of filters is one of the most effective methods to reduce the noise of a signal, improving its fidelity. High and low-pass filters are used to process EMG signals, allowing the low and high-frequency components to be attenuated, respectively. De Luca et al. [37] evaluates the use of HPF to eliminate noise in the low-frequency spectrum, estimating a cut-off frequency of 20 Hz for applications that require common and natural movements or something higher if more vigorous movements are performed, such as those produced in sports activities, but never less than 20 Hz. On the other hand, the use of LPF (Low Pass Filter) with a cut-off frequency between 400 and 500 Hz is recommended. One of the most common solutions used for filtering EMG signals is the use of Butterworth filters, implementing a band-pass filter usually between 20 and 450 Hz.

In pattern classification applications, feature vectors obtained from EMG signals are used as input for classification algorithms. In continuous classification algorithms, adjacent or overlapping segments are used, and therefore, the performance of the system will be affected by window length and allowable delay [38]. Window length determines the amount of information that is used for extraction and classification: more data will produce better results at the cost of increasing the classifier processing time. For a four-state classification system using a majority voting algorithm, Englehart et al. [38] obtain the best results for a window time equal to 32 milliseconds.

### 3.2. Feature Extraction

Once the raw signal obtained from electromyographic sensors has been processed, the next step will be to obtain different features, identifying useful information that is hidden in EMG signals and removing interference or other irrelevant information [39].

The extracted features could be classified in time or frequency domains. Time-domain features represent the energy of an EMG signal, which generally requires low computational load. On the other hand, the features of the frequency domain, in addition to showing the level of activation of the muscles, have the ability to eliminate signal noise but have a higher computational cost [40]. A combination of information from time and frequency domains is defined as Time–Frequency features. The computational load required to obtain features could be a problem in real-time activities. Xiao Feiyun [41] uses the integrated EMG signal obtained through integrated circuits to control an upper-body exoskeleton.

Signals are segmented to extract features of the time domain. Using high segment lengths requires high computational load, while low segment lengths cause biases in the feature extraction. Whilst real-time activities are processed, segments greater than 200 milliseconds require some overlap [42]. Some of the most important features of time domain are Integrated EMG (IEMG), Mean Absolute Value (MAV), Root Median Square (RMS), Simple Square Integral (SSI), or Waveform Length (WL) [39]. Frequency domain features are generally used for the study of muscle fatigue or in motor unit analysis, being not appropriate for the classification of EMG signals [39]. Some of the more important features of frequency domain are Mean Frequency (MNF), Median Frequency (MDF), Mean Power Frequency (MNP), Peak Frequency (PKF) or Total Power (TTP) [39]. 

There is some redundancy in some parameters of time and frequency domains. To avoid data redundancy, Phinyomark et al. [39] evaluate different groups of parameters that obtain good results in movement classification. An example of a group of features used for the classification of EMG signals in the field of myoelectric control is proposed by Hudgins et al. [43]: MAV, WL, ZC (Zero Crossing), and SSC (Slope Sign Change), with a maximum classification precision of 95.67% in the classification of six hand movements [39]. Due to the high number of parameters that can be obtained from EMG signals and other parameters of interest, it is necessary to select the minimum number of parameters that allow obtaining significant results. In order to reduce the number of parameters, Linear Discriminant Analysis and Principal Component Analysis are generally used [44].

### 3.3. Complementary Parameters

For certain applications, it is convenient to use complementary parameters to validate or complement electromyographic signals. The acquisition of these parameters will be carried out through sensors of a different nature placed on the human body, such as inertia or force sensors. The combination of signals makes it possible to overcome some of the obstacles encountered in electromyography applications, improving performance and robustness in processes such as motion prediction [27].

#### 3.3.1. Inertial Measuring Unit (IMU)

The use of inertial sensors for the acquisition of movements represents a reliable alternative to the classical systems of optical classification of movements, being able to reconstruct the movement of various body segments [45]. IMU sensors are usually composed by accelerometers and gyroscopes and could be used to determine velocity, position, and body orientation [46,47]. Lopes et al. [48] evaluate the combination of EMG and IMU sensors for a hand gesture classification process, reducing the error obtained in the segmentation by combining both sensors. 

The use of IMU has some limitations, such as accumulated error, magnetic disturbances, and the need to be calibrated before use. 

#### 3.3.2. Force Sensitive Resistor (FSR)

FSR sensors are used to detect pressure, slip, and weight change. The use of FSR sensors represents a prevalent method for identifying gait phases, presenting fewer errors than other alternative systems. Using two sensors on each foot, which will be placed on the heel and toe, the weight distributions can be analyzed during a gait cycle.

Nazmi et al. [49] use the values obtained using FSR as a reference to train a neural network, subsequently evaluating the convenience of using sEMG signals to identify standing and swing phases during gait. They obtain promising results, so the combination of both types of signals can be used for the development of a robust identification system in gait-related applications. Anvaripour et al. [50] analyze the use of force myography (FMG) of a worker forearm combined with robot kinematics to identify collision between the worker forearm and the robot in a collaborative setup using a Deep Learning techniques. This technique is more flexible to be implemented in industrial settings and tries to identify human applied force processing the forearm muscle movements.

#### 3.3.3. Electroencephalography

Electroencephalographic signals, also called EEG, represent brain bioelectric activity and are related to neuronal activity produced within the brain. There is a variation in the functional integration of brain and muscles when there is a movement intention [51]. However, in rehabilitation applications, the use of EEG signals to control external devices is limited due to low precision, while the use of EMG signals can be limited by the lack of muscle activity due to possible neuromuscular diseases [52]. A mixed system combining brain and muscle activity could improve the usability and reliability of control systems. 

Lange et al. [53] develop a mixed system for classifying the movements of grasping and releasing the hand, using EMG and EEG signals, making it possible to integrate both technologies for applications that require the classification of movements. EEG signals could also be used to identify high-level cognitive states, such as error detection in HMI systems [54].

#### 3.3.4. Electrocardiography

An electrocardiogram, identified as an ECG, is the graphical representation of the electrical activity of the heart as a function of time. Although the activity of the heart usually presents several drawbacks in the acquisition of electromyographic signals due to their overlap, there are procedures to eliminate the noise produced [55]. One of the points of greatest interest in the combination of ECG signals in the process of analyzing the activity of the human body is its relationship with muscle fatigue, mental fatigue [56], or emotional state [57], which are of great interest for human–machine interaction.

### 3.4. Classification

The information extracted from the EMG signals and other parameters is fed into a classifier, which will be used to identify patterns and match results. Several classification methods are used for the analysis of EMG signals. Statistical classifiers, such as Linear Discriminant Analysis (LDA*)* and Principal Component Analysis (PCA), are simpler to implement and require less learning time, which makes them good candidates for real-time application classifiers [40]. Moreover, classifiers such as PCA could be easily integrated with Machine Learning (ML) algorithms [58].

Other classification techniques based in ML are used for gestures and movement identification, obtaining better results than other traditional methods [59,60,61,62], despite presenting some disadvantages such as the lack of transparency in the determination of results [12].

The use of Artificial Neural Networks, ANN, has become a handy tool for predicting and classifying patterns. Originally presented in 1958 by Rosenblatt [63], their greatest potentials are a high processing speed, adaptability, and non-linearity, which makes them very effective, efficient, and successful in problems of both high and low complexity [64]. 

The use of neural networks for the analysis of EMG signals has been proposed as a valid method for myoelectric control for decades compared to other classification methods, such as LDA or kNN (k-Nearest Neighbors); in 1993, Hudgins et al. [43] proposes the use of a multilayer perceptron for the myoelectric control of prostheses. Four contraction movements are performed: contraction of flexor and extensor muscles of the elbow and medial and lateral rotation of the humerus, with EMG sensors positioned according to the characteristics of each user, amputated above or below the elbow. The classification system obtains correct results between 70% and 98% of the test patterns used after the network training period. Nazmi et al. [49] use a multilayer perceptron to assess the phases stance and swing phases of gait. The network diagram of the multilayer perceptron corresponds to its most basic architecture: the input layer, a hidden layer made up of ten neurons, and the output layer. Levenberg–Marquardt (LM) and Scaled Conjugated Gradient (SCG) algorithms are used for the training phase. Seventy percent of the data are used for training, 15% are used for validation, and the remaining 15% are used for testing. Using two sEMG channels positioned in the Tibialis Anterior and Gastrocnemius (middle head) muscles, different sets of temporal domain parameters are evaluated. The test results show an average reliability of 87.4% in the detection of the support and balancing phases for the network trained with the LM algorithm and the RMS, Standard Deviation (SD), MAV, IEMG and WL parameters, demonstrating the capacity of this type of network for the detection of gait phases by electromyography.

Although neural networks applied to electromyography are usually applied in movements and gestures classification, other applications could be found, such as the identification of an ergonomic index related to the user activities. Varrecchia et al. [65] analyze three possible values of lifting index, which are associated with the musculoskeletal risks of a load lifting activity performed by a user. Several multilayer perceptrons trained with six different sets of parameters are used, as well as different combinations of hidden layers and number of neurons.

Deep Neural Networks, DNN, are multilayer perceptron with a complex structure of layers. Its fundamental difference with ANN networks lies into the complexity of connecting each layer and a greater number of neurons, which is why high computing power is required to process complex problems [64]. Mukhopadhyay et al. [60] performed an experimental study to classify hand movements using EMG signals based on deep neural networks. The selection of features affects the reliability of a classification system, but this process could be relegated to deep neural networks itself, which will be fed with the selected parameters without being reduced. Six features are determined for each of the seven channels used, which are concatenated and used to feed the DNN. The classification results obtained are compared with other traditional classification methods. For the classification of eight hand movements, they obtain a classification reliability of 98.88%, 98.66%, 90.64%, 91.78%, and 88.36% for the DNN, SVM (Support Vector Machine), kNN, Random Forests, and Decision Trees classifiers, respectively.

One of the most widely used deep networks is the Convolutional Neural Network, *CNN*, which has a structure adapted to process matrix data, making it especially useful for image processing [66]. The use of CNN networks for the analysis of EMG signals is quite recent. Tao et al. [67] use a convolutional network to identify a worker’s activity using a Myo bracelet, which provides data from an IMU sensor and eight electromyographic channels. The activity maps of the IMU have a 30% higher performance compared to the rest of inputs, having more discriminatory components associated with the activity that the EMG signals. However, the highest performance is obtained by combining the IMU activity maps with the EMG activation vectors, obtaining a 97.6% reliability in activity recognition.

Extreme Learning Machines consists of a network with a single layer connected with random weights to the input layer. Developed by Huang et al. [68,69], ELM (Extreme Learning Machine) classifiers have some properties that make them very useful for real-time applications [26], such as learning speed or high generalization capabilities. Anam et al. [59] analyze several models of ELMs for the control of the hand prosthesis, processing the electromyographic signals of amputees and non-amputees. Non-amputee participants perform 15 different movements with their fingers, while amputee participants simulate 12 movements, imagining they are performing those movements. Twelve channels are used for non-amputee participants, and 11 channels are used for amputee participants to acquire the electromyographic signals, extracting several sets of features. The best classification results are obtained using an RBF-ELM (Radial Basis Function-Extreme Learning Machine) network, reaching a maximum of 99.65% for non-amputated limbs and 99.58% for amputated limbs, compared to other variations of the ELM network and traditional kNN and LDA methods.

Freitas et al. [26] evaluate the use of low-cost open source hardware for the classification of electromyographic signals using ELMs to test the feasibility of controlling vehicle functionalities with EMG. Of the ten hand gestures, seven characteristics of the time domain are processed, using two channels positioned on the forearm. A maximum classification accuracy of 86.53 ± 4.90% is obtained for 200 neurons in the hidden layer. It is interesting to note that for 1000 neurons, lower reliability is obtained in the real-time application during the test, with 79.68 ± 4.10% reliability, so increasing the number of neurons does not mean that the reliability of the classifier system will also improve.

Support Vector Machines are originally developed by Bernhard et al. [70] and Cortes et al. [71] for binary classification problems. Alkan et al. [61] use SVM to classify various arm movements: flexion and pronation of the elbow and pronation and supination of the forearm, comparing the results with the LDA classifier. Using the Mean Absolute Value, MAV, as a parameter, they reach a maximum precision of 99%, which is slightly higher than those obtained by the LDA method: between 96% and 98%.

Hassan et al. [62] used a Myo bracelet, with eight channels taken from the forearm, to classify seven proposed hand movements. The proposed movements are used to control a robotic arm using inexpensive hardware. Several classification methods are compared: SVM, LDA and k-NN, obtaining a classification precision of 95.26%, 92.58% and 86.41% respectively.

Eisenberg et al. [72] evaluate the use of sEMG for monitoring a prosthetic hand in real time, using SVMs for the classification of various hand movements. The sum of the amplitude of the data belonging to three frequency bands is used, obtaining 89.39% reliability in the offline study and 84.93% reliability in real-time.

## 4. Methodology

**Figure 1:**
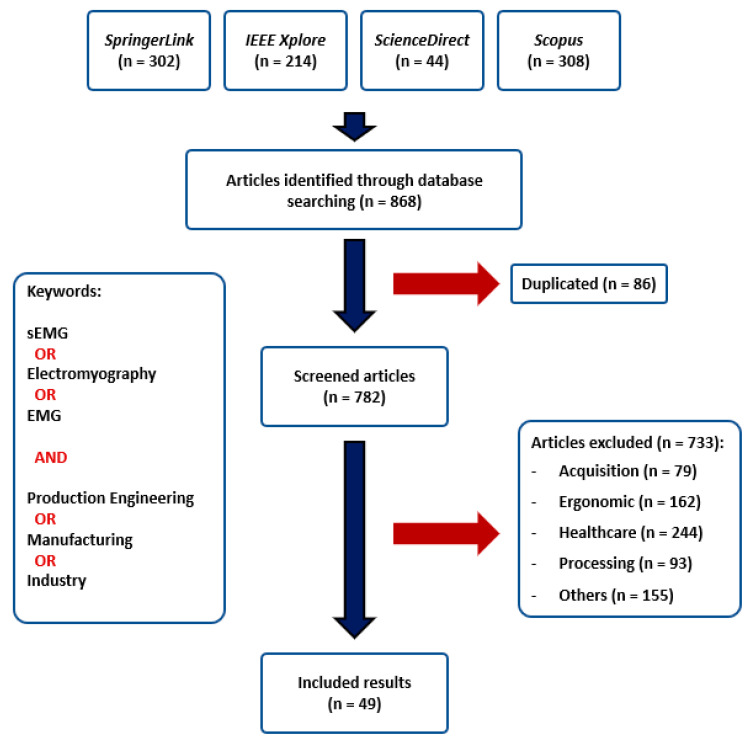
Flow-chart of article identification and screening.

Production engineering is the field of engineering that focuses on the manufacturing processes and activities required for the transformation of raw materials into industrial and consumer goods. To identify the state-of-the-art of EMG applications in production engineering, a systematic review was conducted. See Figure 1.

### 4.1. Database Searching

The literature search was performed between 2015 and 2020 on the following databases: Scopus, SpringerLink, IEEE Xplore, and ScienceDirect. The following keywords were identified from electromyography: *electromyography*, *EMG*, *sEMG*; and from production engineering: *production engineering*, *manufacturing*, *industry*. The articles identified through database searching include selected keywords from areas in the title, keywords, or abstract. The search was limited to papers published in journals, books, and conference proceedings.

### 4.2. Literature Screening

After identifying articles from the selected databases (*n* = 868), duplicates were removed (*n* = 86), and a first screening was conducted to evaluate title and abstract (*n* = 782). The full-text reading was done after the first screening for articles that offered valuable information in the title and abstract. Articles and reviews that present real cases of use in production engineering, whether real or simulated, are selected (*n* = 49). Discarded articles could be classified as *Healthcare* if they evaluate the development of prosthesis and orthosis, augmentation exoskeletons for rehabilitation or other purposes; *Acquisition* was used for those analyzing sensor and electrodes technologies. *Processing* articles investigate about feature selection, classification algorithms, and methods, and finally, *Ergonomic* articles are those assessing ergonomics performance, muscle fatigue, and kinematics estimations not applied to production engineering. Discarded articles classified as *Others* could not be classified in any other category and do not involve an EMG or production engineering research topic. Articles that cannot be accessed are also classified under this category.

## 5. Results: EMG Applications in Production Engineering

Analyzing obtained results, EMG applications in production engineering could be classified into three main groups: Ergonomics, Human–Machine interaction (HMI), and Monitoring. Results classified as Ergonomics include ergonomic assessments of activities, workstations, productive structures, tools, PPEs (Personal Protective Equipments), or exoskeletons carried out using EMG technologies. HMI systems acquire, process, and extract EMG signals to translate this information into control signals of industrial equipment. Finally, monitoring processes allow identifying activity, conditions, and capacities of workers in real-time. The articles analyzed are presented in Table 1.

### 5.1. Ergonomics

The main objective of industrial ergonomics is to optimize industrial workers well-being, ensuring optimal mental and physical conditions to improve overall system performance, reducing risks and improving the quality of working life. EMG could be applied to assess the ergonomic performance of the workplace, activity, tool, PPE, device, and the impact of the use of exoskeletons in the activities of the worker; it is designed to improve the capabilities of a worker in carrying out their daily activities, improving the ergonomics of their workplace, and reducing the muscle fatigue. Ergonomic assessments allow increasing efficiency, productivity, and quality in the industry, making more cost-efficient processes [120].

The main EMG features utilized for the ergonomic assessment of activities in production engineering are RMS, MNF and %MVC (Maximum Voluntary Contraction). Those features are analyzed using statistical techniques such as ANOVA, t-tests, or post-hoc Tukey’s HSD (Honest Significant Differences) to compare the reduction of muscular effort and muscular fatigue. Furthermore, most of the EMG ergonomic assessment studies analyzed are carried out in simulated environments and have a short duration, not comparable to a work shift. See Table 2.

#### 5.1.1. Activities

The ergonomic assessment of activities using electromyography technologies in the industry is mainly focused on the assessment of muscle load, fatigue, and musculoskeletal risks on activities carried out within the aerospace and automotive sectors.

Ergonomic assessment processes are regulated by national and international regulations, which establishes a common criterion to identify the risk associated with an activity. A typical example is the NIOSH equation [121], which was developed to estimate the recommended weight for two-handed lifting tasks based on parameters such as frequency of lifting, quality of grip, or duration of the activity. To identify the associated risk, the Lifting Index is calculated as a quotient between the actual load lifted and the recommended weight limit.

Clip insertion is a frequent activity in the assembly processes of the automotive industry. These small, variously shaped components made of metal, plastic, or composite material can be inserted automatically or manually. Gaudez et al. [73] evaluate different clip inserting and adjusting processes in the automotive industry, determining the muscle load of the upper extremities during activity. None of the analyzed techniques offers better average results than the others. The use of non-motorized tools reduces the muscular load on the dominant limb compared to the insert by hand. The insertion by hand or with power tools decreases the muscular load in the non-dominant limb.

Soewardi et al. [78] analyze muscle efforts of workers during pottery manufacturing, which is one of the strategic industries in Indonesia. The highest contraction is reached in the dorsal lumbar muscle. This muscle has a similar maximal contraction level in both male and female. No differences in muscle contractions are identified between right and left interscapular muscles, even though workers’ posture is not symmetrical.

Overhead activities are widely performed in production environments and are a frequent source of shoulder injuries. These types of activities are identified by requiring the position of hands above the acromion or over 60° shoulder flexion or abduction. Maciukiewicz et al. [79] compared the muscle demands required during unilateral and bilateral overhead drilling operations using EMG data from six muscles of the back and upper limbs: anterior deltoid, middle deltoid, upper trapezius, infraspinatus, supraspinatus, and the erector spinae muscle. Both unilateral and bilateral overhead drilling operations present similar mean EMG signal, having different muscular distribution. In bilateral drilling tasks, where one hand holds the tool and the other is the part to be drilled, there is an increase in the activation of the upper trapezius, which can have implications in scapular dyskinesia, that is, the alteration of position and movements normal scapula in arm movements. However, the bilateral drill configuration reduces muscle demand on the stabilizing muscles of the scapula.

Grazioso et al. [80] analyze muscle synergies of the upper extremities, evaluating different overhead operations of the automotive industry. The authors define synergy as a group of muscles activated jointly by the same control signal, which can be activated in a coordinated way, simultaneously or with a certain delay. The analysis of the existing muscular synergies during movements facilitates the design of exoskeletons, being able to reduce the complexity in the control systems. For the activities performed above the head, they analyze the screwing, drilling and lever movement, and they identify a single muscle synergy that represents more than 98% of total muscle activation.

Chen et al. [81] evaluates a new method for fatigue estimation during disassembling operations. A virtual reality environment with force feedback equipment is proposed to simulate a disassembly process. The evaluation of this process is useful in the initial design phase of industrial products and allows the designer to select the configuration that induces less muscle fatigue.

Injuries and pain in the shoulder could be produced because of the exposure to pushing and pulling activities. Gruevski et al. [74] analyze the effect on upper limb muscle activity in different configurations of simulated pushing tasks. Fifteen participants complete pushing tasks using a dual-track device with two handles used to simulate industrial pushing activities. The study concludes that the anti-phase pushing increases the effort from shoulders and trunk, so this configuration should be avoided while designing repetitive action tasks.

Carpal tunnel syndrome is a condition in which there is excessive pressure on the median nerve of the wrist, which can cause numbness, weakness, and muscle damage in hand and fingers. This syndrome can be caused by repetitive movements of the hand and wrist, as well as the use of vibrating hand tools. Kumar et al. [77] compare the prevalence of carpal tunnel syndrome in shock absorber production industries with different degrees of automation, which is categorized as traditional and semi-ergonomic. The authors identify that this syndrome is presented in workers of both industries who perform repetitive work on the assembly line, with a higher risk of appearing in the traditional factory. In addition, they identify the sEMG-RMS values of the muscles related to this syndrome in both industries.

Guo et al. [75] assess muscle fatigue during manual packaging activities. The experiment evaluates 18 participants that perform the packaging tasks for one hour, evaluating different package size and muscle induced fatigue in the arms, shoulders, and back. The kinematic parameters of the participants are evaluated, validating that musculoskeletal discomfort is prevalent in the upper limb in manual packaging tasks, and it is mainly focused in the arms. Posture changes are also identified, depending on the size of the packages.

Several outdoors and indoors works are exposed to high and low ambient temperatures, which may affect the performance of manual tasks. Renberg et al. [76] evaluate muscle activation and fatigue in upper limb during manual tasks in cold conditions, considered as −15 degrees Celsius in the experiment. Fourteen participants carry out manual overhead and hip level works for five minutes. The experiment concludes that muscle activation level in the forearm was higher in cold conditions.

#### 5.1.2. Workstation

Plant layout is a critical aspect of the efficiency of an industry. This distribution affects all areas, including production lines or different product warehouses. Furthermore, the physical distribution of the workstation has a great impact on a worker’s day-to-day life. Good workstation design reduces the muscular loads and fatigue in operations that are carried out daily. The use of CAD/CAE tools for workstation design and postural assessment could be used to reduce musculoskeletal injuries in productive processes [122]. The use of these tools during workstation design is very useful to identify major ergonomic issues, but the setup required to capture real movements during industrial activities might limit this technology to specific tasks. Wearable technology based in EMG complement and improve traditional ergonomic assessment methods.

One of the parameters that affects the configuration of a workstation is the position and height of parts and tools containers. Lee et al. [82] analyze the effect of the workstation settings on upper body muscles for repetitive and manual transfer operations. The muscular activity of ten seated subjects is evaluated while performing the following tasks: picking connectors located in a drawer positioned at a certain height and transferring to the height of the worktable. After conducting the experiment, Lee et al. conclude that the working height should be lower than the elbow height, being able to prevent or delay fatigue in the upper body limbs.

Antle et al. [83] conducted an experiment to evaluate the impact of using an upright or partially upright posture at the workstation. The experiment consists in performing repetitive box folding tasks, measuring EMG signals and blood pressure after each work activity. After analyzing the data of fifteen participants, they conclude that the semi-upright posture does not offer better muscular performance in the back and upper limbs. Still, it favors the comfort of lower limbs and blood pressure. Ding et al. [84] explore the variation in muscle activity during prolonged sitting work to analyze the effectiveness of different break types. Up to 72 office workers participated in the experiment, 24 of them in the EMG activity measurement activity. The participants carried out computer works during 2 h with different breaks configuration. The study concludes that office workers should have a break after 40 min of sedentary work, whether passive or active ones.

#### 5.1.3. Productive Structure

Electromyography could also be used to identify how productive planning affects the physical condition and productivity of workers. A simple modification on the rotation of activities, diversification of jobs, and the increase of rests periods is widely accepted as a good practice to improve the health of workers without significantly altering the production process [123].

Santos et al. [85] evaluate the influence of rest periods and rotations between workstations on upper body muscle fatigue for repetitive and low-load activities, reviewing relevant articles published. Although they do not identify conclusive results on the impact of rotation on muscle fatigue or worker discomfort, they determine that an increase in work rate shows signs of fatigue in EMG signals, consisting of a lower frequency and increased amplitude.

Bergamin et al. [86] investigated the effect of changing the pace of work and implementing two types of pauses during an assembly task. A simulated activity is carried out for forty minutes, consisting of reaching for an object, manipulating it, and classifying it with the right arm. Different operating rates and pauses are evaluated. The results obtained identify a reduction in muscle load and greater distribution in the activation of the shoulder girdle muscles during lower rhythm assembly operations without being affected by the type of pause, whether active or passive. 

It can be verified that electromyography is an effective tool to ergonomically evaluate the production organization and identify the best possible configuration, being able to improve the health conditions and worker performance.

#### 5.1.4. Tools and PPEs

One of the objectives of the ergonomic evaluation carried out by electromyography is to evaluate the impact of the use of new tools, equipment, or other elements whose objective is to improve the health and safety conditions of workers. The correct identification of the outcome produced on the workers allows selecting the optimal tools or equipment for the production process. Reinvee et al. [90] analyze the ergonomic benefits of an angle grinder with a rotatable handle in a cutting task. Three different postures are analyzed using a dynamometer, EMG, force resistors, and IMU signals. The obtained results favor the less deviated posture from the wrist neutral position, but the muscular load and forces measured do not allow clearly identifying one posture to another, so further investigations need to be carried out.

Wilson et al. [89] measure the force needed to push an industrial cart with different loads and handle heights. They monitor the electromyographic signals obtained from *flexor digitorum*, *deltoid*, and *upper trapezius* with a force sensor located on the handle and the worker´s pulse. Using genetic algorithms based on the obtained data from different subjects, the optimal handle height that causes the minimum compression loads in the L5/S1 vertebral segment turns out to be 109 centimeters. Previous studies had estimated an optimal range for similar conditions between 91 and 112 centimeters. Rashid et al. [91] evaluates push and pull activities in simulated industrial production settings of industrial cart pullers, evaluating EMG signals obtained from various muscles. The results show the seated torso pull as the maximum pull strength posture. The strength values could be used to standardize the load of cart pullers or design handles and flooring.

The hot forging industry has work environments that are very harmful to workers health, such as noise, vibrations, and high temperatures, so workplace incidents occur frequently. Existing industrial processes in this sector cause great fatigue in workers, which is generally due to the transport of heavy products. Song et al. [87] had developed an assistance device that allows compensating the weight of products in the forging industry, evaluating the effectiveness of its use with EMG signals obtained from various muscles of the forearm and hand. A reduction in the mean frequency was identified for elements of 1.5 kg, although without statistical significance. On the other hand, the use of this assistance robot has greater statistical significance for objects weighing 3 kg, obtaining good results in the *extensor digitorum communis*, related to the grip strength of the hand.

The use of safety footwear is necessary for the production area of any industrial plant, so in addition to safety, comfort during its use is a fundamental factor that must be considered. Huebner et al. [88] analyzes the effect of adding interchangeable cushioned heels to the safety shoes. By evaluating EMG signals of twelve muscles of the leg and trunk of various subjects, they conclude that the use of cushioned heels optimizes the energy effort required during movement. These cushioned heels were selected based on the weight of each subject, allowing a reduction in muscle fatigue and musculoskeletal risks.

#### 5.1.5. Exoskeletons

Exoskeletons are devices used in the industry to improve the capabilities of workers, reducing the physical workload required on their activities. According to energy utilization, two different types of exoskeletons could be identified: active or passive. The difference between them is that active exoskeletons use powered actuators, such as electrical, pneumatic, or hydraulic, whilst passive use flexible materials that can store and transmit energy, such as springs. Viable prototypes are beginning to be developed and are suitable to be implemented in a production system, as evidenced by the results obtained from ergonomic assessments during their use.

Bosch et al. [92] analyze the passive Laevo exoskeleton [124]. Simulating an industrial activity of assembly and static load lifting, the kinematics, discomfort, and muscular activation of various muscles involved in the activities carried out, such as the Erector Spinae, the Biceps Femoris or the Trapezius Ascendes, are analyzed. A reduction between 35% and 38% of muscle activity in the lower back region, as well as a reduction in the discomfort of this region, is identified when using the exoskeleton. In addition, three times greater resistance is achieved in static lifting, although there is an increase in discomfort in the chest region. Luger et al. Ref [99] analyze the effect of wearing a passive lower-limb exoskeleton on simulated industrial tasks, such as screwing or clip-fitting. The physical load, kinematics, discomfort, and postural control are evaluated in 45 participants for 21-min simulations. The exoskeleton Chairless Chair™ (Noonee, Deizisau, Germany) is evaluated, and results show a significant reduction in the muscular activity of the lower extremities, which may result in a decreased risk of musculoskeletal disorders of the lower limb. However, the results are not conclusive with regard to the risk of developing back disorders, as the erector muscle activity is reduced, but the trunk flexion angle is increased.

Lee et al. [93] evaluate a passive exoskeleton developed to assist in lifting loads, which is controlled by a finite automaton that modifies its state as a function of lower body angle, angular velocity, and trunk inclination. Studies performed with the exoskeleton identify a reduction in the level of activation of the Erector Spinae and Rectus Abdominis muscles when carrying out a lifting activity, which suggests that the use of this exoskeleton model allows reducing the compression of the spine lumbar during these types of operations.

A comparison between three upper-limb passive exoskeletons is carried out by Alabdulkarim et al. [100], analyzing the physical demand of 12 participants required during mock drilling tasks with different levels of precisions. The participants realized the drilling tasks wearing the Fawcett Exsovest™ (Tiffen Company, Hauppaugen, NY, USA), the EksoWorks™ Vest (Asa Industrial, Alvorada, Brazil), and the FORTIS™ Exoskeleton (Lockheed Martin, Bethesda, MD, USA). These exoskeletons have different designs and are intended to support the upper extremities. Its use could result in an increased physical demand on the lower limb and the reduction of the overall muscular activity in the upper limb. It has been also identified that the use of the proposed exoskeletons might affect the quality of the tasks. A similar study is carried out by Pinho et al. [103] with the upper limb exoskeletons ShoulderX™ by SuitX (Emeryville, CA, USA), Mate™ by Comau (Grugiasco, Italy), and Paexo™ by Ottobock (Duderstadt, Germany). The aim of the investigation is to identify how a tool’s weight and position affect the shoulder muscular activity in simulated overhead industrial activities. The results obtained indicate the beneficial effects in reducing the muscular activity of the upper limb under a simulated industrial setup.

The Hyundai Vest Exoskeleton (H-Vest), a light-weight passive upper arm exoskeleton for overhead tasks, is introduced by Hyun et al. [101]. The proposed exoskeleton uses an adjustable spring mechanism to dissipate energy and generate assistive torque whilst the arm is raised during overhead tasks. The effectiveness of the H-Vest is evaluated using sEMG, identifying a reduction in physical demands of the upper limb during a simulated overhead drilling task. The simulation of industrial tasks to test the effectiveness of exoskeletons might lead to incomplete conclusions, as the exoskeleton is evaluated under very specific activities. Iranzo et al. [102] evaluate the effectiveness of using a passive exoskeleton in the automotive sector in real conditions with 12 participants. The experimental results show a clear reduction in the muscular activity required for arm flexion, reducing the fatigue and discomfort, but they do not reveal a reduction in the risks of injury. The analyzed data and workers feedback allow identifying a slight reduction in the range of back movements. In the study, six operators wore the device 12 h on average, in which 7 h was the shortest time used. Discomfort related to EMG acquisition system is not reported. However, there is not enough information to identify how the exoskeleton usage was distributed.

Chen et al. [94] develop a control system for an active exoskeleton used to facilitate load-lifting activities. This control algorithm is based on the hip and trunk angles and achieves 97.48% ± 1.53% reliability regardless of the subject. The use of sEMG signals for control is not considered because of the difficulty of implementation in an industrial environment. This inherent difficulty is associated with the need to wear electrodes and degradation of the signal due to sweat. The results obtained from the electromyographic sensors suggest an average reduction of the IEMG parameter greater than 30% in the Erector Spinae muscle, analyzing three channels in the thoracic, lumbar, and iliocostal sections, which render the exoskeleton appropriate for lifting activities.

Ogawa et al. [95] designed a low-cost exoskeleton that uses pneumatic actuators to simulate artificial muscles. It includes a control system based on sEMG used to activate or deactivate the exoskeleton by compressing the muscle masseter. Again, a significant reduction in %MVC is observed during the user’s movement, with the study focusing on load-lifting activities.

Thakur et al. [96] propose an active pneumatic exoskeleton for gait assistance, with a control system based on resistive sensors. As previously analyzed in this research work, the weight distribution identified by resistive sensors positioned on the sole makes it possible to evaluate the phases of the gait the user is in, whether supporting or balancing. To evaluate the performance of the system, sEMG sensors are used to determine muscle activation during gait with and without an exoskeleton. As in ergonomic analyses of other exoskeletons, a reduction in the muscle activation of the muscles involved in movement is obtained, achieving a maximum reduction of 44% in the Rectus Femoris muscle with an assistance pressure of 60 kPa.

Otten et al. [97] evaluated the Lucy 2.0 exoskeleton, which is an active pneumatic exoskeleton used to perform overhead activities comfortably. As a result of the difficulties relating to using electromyography as control signals due to its variability, a control system used to increase the support provided through pushing a button is developed. To assess the impact of using the exoskeleton, the muscle activity of the anterior deltoid is measured, which is the muscle that contributes the most to supporting and moving the arm in this type of activity. This study identifies a variation in the activity of this muscle between −13.6% and −49.3%. Furthermore, there is an increase in speed in the upper extremities, which may indicate a positive effect on productivity.

The use of industrial exoskeletons reduces muscle activity and risks of developing musculoskeletal diseases among workers, but there is no standardized methodology to compare their effectivity. Grazi et al. [98] propose a list of possible metrics to compare load-lifting exoskeletons.

### 5.2. Human–Machine Interactions

A human–machine interaction (HMI) system must be able to acquire, process, and extract data from electromyography signals. Afterwards, these data should be translated into control signals that will be used to adapt the operation of industrial equipment. Within this sector, it is possible to identify two clear sections: myoelectric control systems and human–machine cooperation systems. The first group seeks to replace the traditional equipment control channels through new interfaces based on EMG, providing a more intuitive and effective control system. The analyzed experimental studies are carried out in simulated environments and combine EMG signals with other control systems and equipment, such as the HoloLens VR Kit [106] or an industrial joystick [107]. See Table 3 and Table 4.

On the other hand, HMI cooperation systems use EMG to integrate the human body into the production system, being able to provide information on the movement or muscular fatigue to optimize a collaborative activity. Some examples of the collaborative activities analyzed are supervisory control, fatigue adaptation, hand-over activities, and motion prediction. Artificial intelligence processing techniques are widely used to classify information obtained from EMG acquisition systems, which can be combined with ECG signals [54] or augmented reality kits [114]. The analyzed studies are carried out in simulated environments.

#### 5.2.1. Myoelectric Control Systems

The development of natural control strategies represents one of the significant challenges of human–machine interface design. In this sense, myoelectric control seeks to replace traditional control channels through new interfaces based on electromyography, being able to provide a more intuitive and efficient control system integrated with the user’s movements. See Table 3.

Being able to control a robot remotely is crucial in industrial environments where the physical integrity of the worker is at risk. Furthermore, the use of technologies such as virtual reality allows the operator to interact in different ways with the environment in real time. Wu et al. [106] explore a mixed virtual reality and electromyography control system to operate a robot remotely. A Myo device is used to acquire the electromyographic signals in addition to a Microsoft HoloLens virtual reality device. Hand gestures control the speed of the robot, while the orientation is controlled with the virtual reality glasses, establishing a straightforward and natural control system. Palar et al. [107] introduce a novel HMI control system to adapt the autonomy of a climbing robot used for inspecting weld beads in storage tanks according to the operator´s experience. This robot operates at temperatures close to 100 degrees Celsius during inspections. The operator uses an industrial joystick complemented with a Myo armband that contains IMU and sEMG sensors. The levels of autonomy manual, shared, supervisory, and autonomous modes are identified. Up to five participants with different skills levels operate the robot and tried to inspect the weld beams of the vessel. After analyzing the information, it is verified that the alignment error is very similar despite operators’ abilities, so the system could adapt properly to the operator.

Some of the gripping devices used in the industry have unintuitive control systems that have no relationship between the handheld system and the action produced. Thus, a tailored control system could improve the overall usability of the system. Meattini et al. [105] explore a control system for gripping equipment using surface electromyography. The designed system is composed of eight sEMG channels positioned on the forearm and an SVM classification system, which allows the movement of the user’s hand to be identified with the gripping devices of this study. Finally, system reliability equivalent to 96.3% is obtained in healthy subjects, defining a more natural control system for this type of device.

One of the limitations of the implementation of electromyography in industrial environments is the displacement of the electrodes in workers due to their activities. Fu et al. [104] evaluate the acquisition of signals in unfavorable positions of the muscle, as well as the contribution of electrical noise from industrial equipment in the acquired signal. Although noise is evident in the signal due to the operation of the equipment, it does not represent a significant alteration in its amplitude. They conclude that the position of the electrodes is critical to obtain correct measurements.

Despite having achieved great advances in the laboratory, the real applications of pattern recognition for myographic control do not have significant improvements. One of the reasons behind this limitation is the lack of two-way interaction between the user and the device. In the extremities of the human body, touch works as a feedback loop that helps control muscle contraction during movement [27]. In the field of exoskeleton control to improve the capacity of workers, control systems based on electromyography present advantages over other alternative methods, at the cost of higher computing requirements and other inherent problems, such as the use of electrodes, the signal variability, or signal processing [108].

#### 5.2.2. Human–Machine Collaboration Systems

With the automation of production processes, it becomes necessary that an effective human–machine communication system is integrated, allowing workers to interact efficiently and safely with industrial equipment. The cooperation between people and robots allows combining the cognitive capacity of the human being with the physical power of a machine [110], defining an exciting area for the industrial field that allows optimizing a collaborative activity. See overview in Table 4.

The collaborative manipulation of tools requires an adaptive two-way interaction between human and machine, understanding the intentions of the collaborator and being able to anticipate their movements, as well as communicate autonomously and adapt to the requirements of the activity. Wang et al. [112] analyze the process of object transfer between humans and machines in collaborative environments. During an activity of these characteristics, it is important that the robot understands and anticipates the intention of the human being. To identify this information, a Myo band is used, which has an IMU sensor and eight sEMG channels positioned on the user’s forearm. The position and posture of the forearm is transmitted to the robot, which recognizes and interprets the intentions of the collaborator to adapt its operation, such as “I need this object from you” or “I need to give you this object.” A collaborative assembly activity is simulated. Although the system does not reach 100% reliability, it is highly effective and adds advantages to the process, allowing the transfer to be controlled naturally and even accepting or rejecting the object that is being delivered to the user. Techniques of human motion prediction are also analyzed by Tortora et al. [113], introducing a real-time classification system with a 94.3% ± 2.9% of accuracy for human–machine industrial systems, and Sirintuna et al. [114], developing a collaboration system adapted to path following tasks.

Peternel et al. [111] develop a cooperative method where the machine adapts to users fatigue, which is identified using superficial electromyography of the muscles involved in the activity. Experimentally, both collaborative sawing and polishing activities are simulated, with the tools being manipulated jointly by the robot and the user. The fatigue pattern is determined by the variation suffered by the signal during activity, observing a change in amplitude and frequency when fatigue increases. Parameters such as recovery rate and fatigue capacity are incorporated to model muscle behavior. When the activity begins, the robot participates as a follower, learning (strength, trajectory, etc.) until it can perform the activity collaboratively. Once the established fatigue threshold is reached, the robot increases its participation in the aspects of the activity that it can perform autonomously, while the user continues to perform activities of a collaborative nature, such as stabilizing the tool. This method considerably reduces the muscular effort of the user, being able to partially relax and recover once the robot takes over the physical execution of the activity.

Similarly, Huang et al. [109] conducted a study on a cooperative sawing activity, simplifying the activity into a push phase of the participant and a second push phase of the robot. To optimize collaborative activity, the robot must know the phase of movement in which the collaborator is, for which six electromyographic channels are used in various muscles of the upper body. The backward propagation methods LDA, SVM, and ANN are used as classifiers, obtaining a maximum reliability of 90% in collaborative activity. Reference is made to the fact that the selected parameters represent only a part of the human movement information, so a 100% reliability can never be achieved.

The use of biosignals such as electromyography (EMG) and electroencephalography (ECG) can be used to anticipate human intention in a collaborative environment. One of the most significant uses of these signals is the estimation of the physical and mental state of the user, being able to evaluate muscle fatigue, inattention, or anxiety [110].

DelPreto et al. [54] use a mixed robotic arm control system to assess hypothetical risk situations: a user monitors the activity of a robotic arm that simulates a drilling operation. A perception of error is identified from the EEG signal, which causes the robot to stop immediately to request assistance. Finally, the user makes a hand gesture to make changes to the drilling position of the robot. 

It seems clear that the development of this technology can be applied in the industrial field to improve worker safety in an automated environment and can also modify the operating modes of each equipment.

### 5.3. Monitoring

Knowing the activity, conditions, and capabilities of the worker during the performance of their work allows the optimization of a production process, evaluating the performance of the worker and avoiding or preventing musculoskeletal risks. The analyzed experimental studies require both muscular assessment and motion reconstruction, so EMG acquisition techniques are often combined with optical or inertial motion analysis systems, such as the SMART-DX 6000 System (BTS Bioengineering, Quincy, MA, USA) [65] or Vicon motion capture system (Vicon, Yarnton, UK) [117]. Human body modeling can be used in combination with motion capture systems and FSR sensors to identify muscle or joint contact force [116]. Finally, with regard to IMU motion reconstruction techniques, the Myo armband is commonly used because it captures both EMG and IMU signals [67,117,119]. As identified before, most of the studies are carried out in simulated environments and have a short duration.

Occupational musculoskeletal injuries are often caused by common movements, such as lifting weights, grasping movements, or intense typing, which can become risky movements if performed too frequently and without rest (see summary in Table 5). Peppoloni et al. [115] propose a portable system that allows the evaluation of biomechanical risk in repetitive efforts. The system communicates via Bluetooth with a computer where the data is processed, which is obtained by inertial sensors positioned on the arm, forearm, and hand, along with eight sEMG channels. The activities carried out are recorded and evaluated by two experts, identifying the RULA (Rapid Upper Limb Assessment) and SI (Strain Index) indices. The results obtained after the signal processing reach a 94.79% reliability for the RULA index and a 44.79% for the SI index.

Recording systems or motion acquisition systems have been traditionally used to identify the activity of an individual, both having great reliability in recognizing gross movements. However, some industrial processes may require the identification of activities in a much more detailed way, such as the analysis of hand or finger movements, so the use of alternative systems is necessary. 

Kubota et al. [117] compare the effectiveness of a motion acquisition system with a system developed using surface electromyography and inertial sensors. The author evaluates various activities carried out in the automotive industry and process data obtained with various classification methods: SVM, LDA, and kNN. Complementary benefits are identified in the use of both systems: capturing general movements is done more effectively with the motion analysis method, while more detailed movements are better identified with the system based on electromyography and inertial sensors.

Tao et al. [67] develop a system for evaluating worker activities using inertial sensors and a Myo sEMG signal acquisition device. Data are acquired from eight subjects who perform six different activities, such as removing a tool from a holder, hammering, or tightening nuts. These data are processed with a Convolutional Neural Network (CNN), reaching up to 98% accuracy in the identification of activities. Al-Amin et al. [119] complement IMU and EMG signals obtained by two Myo-armbands with a vision based sensor, Microsoft Kinect, which is used to track skeletons joints of the workers. The collected data are used to train different CNN models. The study shows that IMU and EMG signals perform well in recognizing fine movements, while the skeletal data perform better recognizing coarse activities. Hence, the fusion method is effective to improve the accuracy of the system by complementing information and overcoming the limitations of each system.

Muscular overload is a critical aspect of workers’ health in the industry, and it has been widely studied using observational methods. Greco et al. [118] develop a quantitative procedure to assess the risk of biomechanical overload using surface electromyography and a motion capture system based on inertial sensors, evaluating the potential of using the Internet of Things. A Raspberry is used to acquire and process the data obtained from six sEMG channels positioned on the trunk and eight inertial sensors, arranged on the upper extremities. The study subject performs different assembly activities in the gearbox area of a production line in the automotive industry. The use of this system is compatible with the worker’s activity, without presenting interference between the signals sent by the devices and plant’ equipment assessing the biomechanical load of the worker in real-time. However, future studies may be required to validate the proposed method.

In addition to the recognition of activities, one of the topics that arouse the greatest interest in the research is the lifting and transporting of loads, as they are a frequent source of musculoskeletal incidents and disorders. Li et al. [116] evaluate muscle activity during repetitive manual load carrying, identifying electromyographic signals obtained from the muscles of the lower back, which are the muscles most involved in this type of activity. The experiment carried out does not have the intensity necessary to identify the effect of fatigue on the acquired sEMG signals, concluding that the effectiveness of this technology is limited to superficial muscles and areas with reduced adipose tissue.

Varrecchia et al. [65] identify the Erector Spinae Longissimus muscle as the muscle of the trunk most sensitive to variations in lifting conditions, obtaining up to 89.1% reliability in the identification of the Lifting Index, LI = 1 index and a 76.4% reliability for the LI index = 3, which is the one with the highest risk. The best results are obtained with an artificial neural network with three hidden layers and fifty neurons in the first layer. The authors identify that better results could be obtained with a more complex network architecture. The integration of artificial intelligence with surface electromyography and other signals allows automating the ergonomic evaluation process of a worker, adapting to traditional evaluation techniques, such as RULA analysis or the Lifting Index. The use of innovative technologies for biomechanical risks assessment at work is at its initial stage. It is mainly based in sEMG, IMUs, and dynamometers sensors [125], and its evolution will allow monitoring the status of workers in real-time as well as the activities they are performing without interfering with them. 

## 6. Conclusions

Electromyography has some limitations that prevent wider implementation in the industry. However, recent technological advances and synergies with other technologies might contribute to the development of new and more reliable applications in the future. Three main fields of application of EMG within production and manufacturing environments have been identified: ergonomics, human–machine interaction, and monitoring.

The main objective of industrial ergonomics is to optimize industrial workers well-being, ensuring optimal mental and physical conditions to improve overall system performance, reducing risks, and improving the quality of working life. EMG could be applied to assess the ergonomic performance of the workplace, activity, tool, PPE, device, or the impact of the use of exoskeletons. Compared to traditional methods, such as RULA, EMG ergonomic assessment techniques and portable acquisition equipment allow personalizing the process to each worker and activity.

A human–machine interaction (HMI) system must be able to acquire, process, and extract data from electromyography signals. These signals, combined with other useful information, is used to generate controls signals to adapt the operation of industrial equipment. Myoelectric control systems seek to replace or complement traditional equipment through new interfaces based on EMG. For example, the remote control of robots is crucial in industrial environments where the physical integrity of the worker is at risk. HMI cooperation systems aim to optimize collaborative activities. With the automation of production processes, it becomes necessary that an effective human–machine communication system is integrated, allowing workers to interact efficiently and safely with industrial equipment. The use of EMG signals and other useful information allows the supervision of robots, motion prediction, or live adaptation to workers capabilities, combining the cognitive capacity of workers and physical power of robots.

Finally, monitoring applications of EMG intend to identify the activity, conditions, and capabilities of the worker during the performance of their work. Generally, muscular assessment will be complemented with motion reconstruction techniques, whether optical or inertial. Muscle fatigue, workers activities, and musculoskeletal risks could be monitored using the combination of these technologies.

EMG ergonomics applications are the most widely and successfully used in industry, as laboratory conditions could be replicated in the industrial environment. Its success relies on the possibility of obtaining conclusive results without affecting workers continuously. Under laboratory conditions, different monitoring and HMI applications could achieve more than 95% of accuracy in real-time classification applications but are not suitable for daily use within an industrial environment. Some of those limitations that prevent the daily use of EMG technologies are the placement of sensors, skin preparation, or training periods between use. New technological development in acquisition methods, with higher usability compared to traditional wet electrodes, will allow using EMG in an industrial environment overcoming some of its major drawbacks.

Most of the production engineering EMG applications reviewed in this paper are carried out in simulated environments and have a short duration that is not comparable to a work shift. Major drawbacks could appear using current acquisition systems daily in real environments. The continuous use of acquisition systems might be necessary for some of the analyzed HMI and monitoring applications, and most of the current EMG acquisition techniques require skin preparation to improve the signal. New data acquisition techniques and tools should be applied to reduce issues related to sensor misplacements and daily setups, such as higher-density EMG sensors embedded in Personal Protective Equipment.

Furthermore, reviewed investigations tend to use a limited number of subjects and laboratory conditions; thus, it will be interesting to test a higher number of users and develop new data processing techniques adapted to an industrial environment, identifying if a setup is feasible and improving the generalization capability of classification methods. Decentralized cloud data processing could be used to integrate different biological signals, such as ECG, EEG, and EMG, with personal IMU signals of all workers from an industrial area, as it has been identified that its combination improves the accuracy of classification methods, and the availability of the data could be used to develop new interesting technologies.

Synergies between EMG industrial applications have been identified, such as the combination of real-time fatigue monitoring, musculoskeletal risk assessment, and assisted handling devices. Different investigations carried out in those EMG applications obtain high reliability under laboratory conditions and use EMG data obtained from the same muscles. To analyze the future integration of EMG applications, it is interesting to analyze the reliability of different EMG applications with the same data, so that a common data source could be used for different purposes.

## Figures and Tables

**Table 1:** Applications of electromyography in production engineering.

Reference	Area	Subcategory	Assessment
[73]	Ergonomics	Activities	Clip fitting methods.
[74]	Ergonomics	Activities	Upper limb muscle activity during pushing tasks.
[75]	Ergonomics	Activities	Upper limb muscle fatigue in repetitive packaging tasks.
[76]	Ergonomics	Activities	Effect of working position and cold environment.
[77]	Ergonomics	Activities	Prevalence of Carpal Tunnel Syndrome in shock absorber production industry.
[78]	Ergonomics	Activities	Muscle contractions on pottery manufacturing process.
[79]	Ergonomics	Activities	Muscle fatigue during overhead drilling operations.
[80]	Ergonomics	Activities	Upper limb muscle synergies during overhead operations.
[81]	Ergonomics	Activities	Muscle fatigue estimation in virtual reality environment.
[82]	Ergonomics	Workstation	Upper body activities during manual transfer activities.
[83]	Ergonomics	Workstation	Vascular and muscular discomfort using a sit-stand tool.
[84]	Ergonomics	Workstation	Muscular activity and discomfort during sitting work.
[85]	Ergonomics	Productive Structure	Influence of rest periods and rotations on fatigue.
[86]	Ergonomics	Productive Structure	Pause and pace evaluation during an assembly task.
[87]	Ergonomics	Tools and PPEs	Assistance handling device in hot forging industry.
[88]	Ergonomics	Tools and PPEs	Effects of heel cushioning elements in safety shoes.
[89]	Ergonomics	Tools and PPEs	Push strength of industrial cart pullers.
[90]	Ergonomics	Tools and PPEs	Ergonomic assessment of rotatable handle in angle grinder.
[91]	Ergonomics	Tools and PPEs	Isometric pulls strength of industrial cart pullers.
[92]	Ergonomics	Exoskeletons	Laevo exoskeleton assessment.
[93]	Ergonomics	Exoskeletons	Passive exoskeleton for load lifting assessment.
[94]	Ergonomics	Exoskeletons	Active exoskeleton for load lifting assessment.
[95]	Ergonomics	Exoskeletons	Low cost pneumatic exoskeleton evaluation.
[96]	Ergonomics	Exoskeletons	Pneumatic exoskeleton for gait assistance.
[97]	Ergonomics	Exoskeletons	Lucy 2.0 exoskeleton assessment.
[98]	Ergonomics	Exoskeletons	Lumbar exoskeleton assessment in industrial applications.
[99]	Ergonomics	Exoskeletons	Passive lower limb exoskeleton in industrial tasks.
[100]	Ergonomics	Exoskeletons	Passive exoskeletons comparison in a mock drilling task.
[101]	Ergonomics	Exoskeletons	Passive upper arm exoskeleton for overhead tasks.
[102]	Ergonomics	Exoskeletons	Passive exoskeleton in an automotive assembly plant.
[103]	Ergonomics	Exoskeletons	Commercially available exoskeletons comparison.
[104]	HMI	Control systems	Non-ideal electrode placement for machine control.
[105]	HMI	Control systems	sEMG base HMI for robotic hands.
[106]	HMI	Control systems	Mixed virtual reality and EMG control system.
[107]	HMI	Control systems	Robot autonomy adaptation according to operator skill.
[108]	HMI	Control systems	Control strategies of lower limb exoskeletons.
[54]	HMI	Collaboration	Risk situation identification during drilling operation.
[109]	HMI	Collaboration	Cooperative sawing activity.
[110]	HMI	Collaboration	Human robot collaboration.
[111]	HMI	Collaboration	Robot adaptation to human physical fatigue.
[112]	HMI	Collaboration	Hand-over human robot collaboration.
[113]	HMI	Collaboration	Human motion prediction with wearable interface.
[114]	HMI	Collaboration	Human motion prediction.
[115]	Monitoring	-	Portable system for biomechanical risk evaluation.
[116]	Monitoring	-	Evaluation of muscle activity during load carrying.
[67]	Monitoring	-	Activity recognition in industrial tasks.
[117]	Monitoring	-	Motion acquisition system effectiveness.
[118]	Monitoring	-	Activity recognition in industrial tasks.
[119]	Monitoring	-	Activity recognition in manufacturing assembly task.

**Table 2:** Overview of electromyography (EMG) ergonomic assessment experimental setups in production engineering.

Ref.	Subjects	Environment	EMG Acquisition	Duration
[73]	11M	Simulated	Wave wireless EMG, Cometa Ambu^®^ BlueSensor N-00-S/25	≈3 min
[74]	15F	Simulated	Bipolar surface electrodes, Mitrace	≈50 min
[75]	10M, 8F	Simulated	Biopac MP150 Disposable electrode patches, Xunda	≈1 h
[76]	14M	Simulated	Dual electrodes Ag/AgCl, Noraxon	≈2 h
[77]	140	Real	Biopac MP45	≈10–40 s
[78]	8M, 7F	Simulated	EMG ME3000P8, Mega Electronics	≈20 min
[79]	22M	Simulated	Noraxon T2000 Dual electrodes Ag/AgCl, Noraxon	≈40 min
[80]	1M	Simulated	FREEEMG 1000	≈3 min
[81]	8M, 1F	Simulated	BIOPAC MP150	≈5 min
[82]	10M	Simulated	BIOPAC MP150	≈6 min
[83]	8M, 7M	Simulated	Noraxon	≈34 min
[84]	36M, 36F	Simulated	ErgoLAB, Kingfar Pre-gelled disposable AgCl, Kangren	≈2 h
[86]	18F	Simulated	Delsys Myomonitor IV, Delsys	≈40 min
[87]	3	Simulated	Bagnoli™ Desktop Systems, Delsys	Not specified
[88]	10M	Simulated	Ag/AgCl electrodes H93SG, Covidien	Not specified
[89]	5M	Simulated	Precision bipolar EMG sensors SX230, Biometrics	Not specified
[90]	11M	Simulated	BITalino telemetric microcontroller, PLUX Wireless Biosignals Dual electrodes Ag/AgCl, Noraxon	≈1 min
[91]	20	Simulated	IXTA Data Acquisition System	≈12 s
[92]	9M, 9F	Simulated	Porti 16/ASD system, TMS Bipolar Ag/AgCl, Medicotest	Not specified
[93]	1M	Simulated	TeleMyo 2400R, Noraxon	≈1 min
[94]	7M	Simulated	TeleMyo 2400R, Noraxon Pre-gelled bipolar Ag/AgCl surface electrodes, Pirrone & Co.	Not specified
[95]	1M	Simulated	EMG logger Ⅱ, Oisaka Electronics Co.	≈5 s
[96]	7	Simulated	Personal EMG (P-EMG), Oisaka electronic Ltd.	Not specified
[97]	8	Simulated	ProEMG, Myon AG	≈13 min
[99]	46M	Simulated	Pre-gelled Ag/AgCl surface electrodes, Kendall™	≈2 min
[100]	7M, 5F	Simulated	TeleMyo 900, Noraxon Bipolar Ag/AgCl electrodes, AccuSensor	≈3 h
[101]	10M	Simulated	Trigno Wireless EMG system, Delsys	≈9 min
[102]	11M, 1F	Real	UltiumTM EMG, Noraxon	≈12 h
[103]	2M	Simulated	Myotrace 400, Noraxon	Not specified

**Table 3:** Overview of EMG human–machine interaction (HMI) myoelectric control systems experimental setups in production engineering.

Ref.	Subjects	Environment	Signals	Processing
[104]	1	Simulated	EMG	SNR analysis
[105]	4M	Simulated	EMG	SVM
[106]	-	Simulated	EMG, IMU, VR Kit	Embedded (Myo/HoloLens)
[107]	5	Simulated	EMG, IMU, Industrial Joystick	Fuzzy

**Table 4:** Overview of EMG HMI collaboration systems experimental setups in production engineering.

Ref.	Subjects	Environment	Signals	Collaboration	Processing
[54]	7	Simulated	EMG, ECG	Supervisory control	ANN
[109]	3M	Simulated	EMG	Supervisory control	LDA, SVM, BPNN ^1^
[111]	4M	Simulated	EMG, IMU (Myo band)	Fatigue adaptation	-
[112]	Training (5M, 1F) Testing (4M, 2F)	Simulated	EMG, IMU (Myo band)	Parts hand-over	DAG-SVM ^2^, HMM ^3^
[113]	3M, 1F	Simulated	EMG, IMU (Myo)	Motion prediction	GMM ^4^, PFT ^5^, FDA ^6^
[114]	Training (1M, 1F) Testing (3M, 3F)	Simulated	EMG, AR Kit	Motion prediction	ANN

^1^ Back-Propagation Neural Networks. ^2^ Direct Acyclic Graph-Support Vector Machine. ^3^ Hidden Markov Model. ^4^ Gaussian Mixture Model. ^5^ Probabilistic Flow Tubes. ^6^ Fischer´s Discriminant Analysis.

**Table 5:** Overview of EMG monitoring experimental setups in production engineering.

Ref.	Subjects	Environment	Monitoring	Signals	Motion Reconstruction	Duration
[115]	7M, 3F	Simulated	WMSD ^1^ risk	IMU, EMG	IMU	Not specified
[116]	3M	Simulated	Muscle fatigue	EMG	Optical, Human model	≈7 min
[67]	8	Simulated	Activity (*n* = 6)	IMU, EMG	IMU + EMG	Not specified
[117]	3M, 2F	Simulated	Activity (*n* = 9)	IMU, EMG	Optical, IMU	Not specified
[118]	1M	Real	WMSD risk	IMU, EMG	Optical, IMU	≈2 min
[119]	5	Simulated	Activity (*n* = 7)	IMU, EMG	Optical, IMU	≈6 min
[65] ^2^	10M	Simulated	WMSD risk	IMU, EMG	Optical	Not specified

^1^ Work-related Musculo-Skeletal Disorders. ^2^ Not originally identified in database searching.
